# Identification and validation of a lipid metabolism gene signature for predicting biochemical recurrence of prostate cancer after radical prostatectomy

**DOI:** 10.3389/fonc.2022.1009921

**Published:** 2022-10-17

**Authors:** Yingxin Cai, Jingwei Lin, Zuomin Wang, Yuxiang Ma, Jinyou Pan, Yangzhou Liu, Zhigang Zhao

**Affiliations:** Department of Urology & Andrology, Minimally Invasive Surgery Center, Guangdong Provincial Key Laboratory of Urology, The First Affiliated Hospital of Guangzhou Medical University, Guangzhou, Guangdong, China,

**Keywords:** prostate cancer, TCGA, MSKCC, biochemical recurrence, lipid metabolism

## Abstract

**Background:**

Pro5state cancer is one of the most commonly diagnosed cancers in men worldwide and biochemical recurrence occurs in approximately 25% of patients after radical prostatectomy. Current decisions regarding biochemical recurrence after radical prostatectomy are largely dependent on clinicopathological parameters, which are less accurate. A growing body of research suggests that lipid metabolism influences tumor development and treatment, and that prostate cancer is not only a malignancy but also a lipid metabolism disease. Therefore, this study aimed to identify the prognostic value of lipid metabolism-related gene signaling disease to better predict biochemical recurrence and contribute to clinical decision-making.

**Methods:**

Expression data and corresponding clinical information were obtained from The Cancer Genome Atlas (TCGA) database and the MSKCC database. Candidate modules closely associated with BCR were screened by univariate and LASSOcox regression analyses, and multivariate Cox regression analyses were performed to construct gene signatures. Kaplan-Meier (KM) survival analysis, time-dependent subject operating curves (ROC), independent prognostic analysis, and Nomogram were also used to assess the prognostic value of the signatures. In addition, Gene Ontology Analysis (GO), Kyoto Encyclopedia of Genes and Genomes (KEGG), and Gene Set Enrichment Analysis (GSEA) were used to explore potential biological pathways.

**Results:**

A 6-gene lipid metabolism-related gene signature was successfully constructed and validated to predict biochemical recurrence in prostate cancer patients. In addition, we identified the 6-gene signature as an independent risk factor. Functional analysis showed that lipid metabolism-related genes were closely associated with arachidonic acid metabolism, PPAR transduction signaling pathway, fatty acid metabolism, peroxisome, and glycerophospholipid metabolism. Prognostic models were associated with immune cell infiltration.

**Conclusion:**

We have successfully developed a novel lipid metabolism-related gene signature that is highly effective in predicting BCR in patients with limited prostate cancer after RP and created a prognostic Nomogram. Furthermore, the signature may help clinicians to select high-risk subpopulations, predict patient survival, and facilitate more personalized treatment than traditional clinical factors.

## Introduction

According to statistics, prostate cancer is the second most common cancer and the fifth leading cause of cancer death in men (1, 2). According to the American Cancer Statistics projections, there will be 268,490 new prostate cancer diagnoses and 34,500 deaths from prostate cancer in the United States in 2022 (1). Following the prostate cancer treatment guidelines, radical prostatectomy remains the primary treatment for clinically limited prostate cancer, but biochemical recurrence still occurs in 20% to 40% of patients (3, 4). Biochemical recurrence implies an increased risk of metastatic and Castration-Resistant Prostate Cancer (5). Therefore, it has become urgent to explore the gene expression associated with biochemical recurrence in prostate cancer and to develop a guiding tool to predict biochemical recurrence in prostate cancer.

As a fundamental component of membranes, lipids are the fuel that drives high-energy processes and plays a key role as signaling molecules and regulators of many cellular functions. As research into lipids has intensified, more and more studies have shown that cancer cells have characteristic alterations in lipid metabolism (6). The development of prostate cancer has also been shown to be closely linked to lipid metabolism, and studies have even shown a higher abundance of many classes of lipids in BCR patients, including triglycerides, lysophosphatidylcholine, phosphatidylethanolamine, phosphatidylinositol, diglycerides, acylcarnitine, and ceramides (7, 8). Lipid metabolism-related genes play a key role in this, and increased expression of LPCAT1 is associated with early PSA recurrence after prostate cancer surgery; *SMPLD3B is* highly overexpressed in PCa tissue and negatively correlates with local PCa prognosis (9, 10).

Studies have shown that immune cells in the tumor microenvironment (TME) also undergo lipid reprogramming, for example, the key to activation of regulatory T cells is changes in lipid metabolism (11, 12). At present, studies have shown that abnormal lipid metabolism has an important impact on the molecular mechanism of immune cell function (13). Targeting genes associated with tumor and immune lipid metabolism may have a critical impact on cancer prevention and treatment (14). Therefore, the relationship between aberrant lipid metabolism and tumor immunity is gradually becoming a hot topic of research.

In this study, a series of bioinformatics approaches were used to systematically analyze the expression and potential functions of lipid metabolism genes associated with the biochemical recurrence of prostate cancer. A prognostic signal based on six lipid metabolism-related genes was constructed and validated, which accurately predicted the biochemical recurrence of prostate cancer. In addition, this prognostic signal was an independent prognostic indicator and correlated with immune cell infiltration. These results suggest that lipid metabolism could be a more accurate predictor of the biochemical recurrence of prostate cancer.

## Materials and methods

### Patient and mRNA data

This study included two separate cohorts of prostate cancer patients from the TCGA database and the MSKCC database, respectively. The prostate cancer cohort from the TCGA database contained 411 patients with FPKM data and complete biochemical recurrence status and time, which were randomized 1:1 into a training group (207 patients) and an internal validation group (204 patients). The MSKCC prostate cancer patient cohort from the Cbioportal database was used as the external validation set, containing 131 patients with complete expression profiles (normalized log2mRNA expression data) and clinical information.

### Genes related to lipid metabolism

Lipid metabolism-associated genes were screened by the Gene Enrichment Analysis (GSEA) database and the Kyoto Gene and Genome Encyclopedia (KEGG) database. A total of 931 genes were extracted from 12 lipid metabolism-associated gene sets from the GSEA database and 736 genes were extracted from 15 associated gene sets from the KEGG database. After removing duplicate genes, a total of 1104 lipid metabolism genes were identified for further study.

### Identification of differential expression of genes related to lipid metabolism

Total samples from the prostate based on the TCGA database were used to screen for lipid metabolism-related genes differentially expressed between PRAD and normal samples using R’s Limma package. False discovery rate (FDR) < 0.05 and |log2-fold change (FC)| > 0.5 were set as cut-off criteria.

### Enrichment analysis of differential genes

To further investigate the potential molecular mechanisms involved in differential genes related to lipid metabolism, gene ontology (GO) and pathway enrichment analyses were performed on the above differential genes using the cluster profile R package. FDR values <0.05 were considered statistically significant.

### Protein interaction network construction

Lipid metabolism-related differential genes were submitted to the string database for PPI information and visualized by Cytoscape.

### Construction and validation of a genetic model of lipid metabolism associated with biochemical relapse

Based on clinical data from the training group of the TCGA database prostate cancer patient cohort, the above-screened differential genes were screened for genes associated with biochemical recurrence of prostate cancer using univariate cox regression and lasso regression analysis. Finally, based on the results of the LASSO regression analysis, an optimized risk score model was constructed using the same multivariate cox regression model. Risk scores were calculated by the following equation.


Risk score = (Coef1*expression mRNA1)+(Coef2*expression mRNA2)+ (Coef n*expression mRNA n)

where Coef is the cox regression model coefficient for the relevant mRNA. The median risk score was used as a cut-off value to distinguish between patients at high and low risk of biochemical recurrence of prostate cancer.

The TCGA dataset training and validation groups were used to assess and validate the prognostic model, respectively, and the MSKCC cohort was used as an external validation set to validate the risk score model. log-rank and Kaplan-Meier (K-M) tests were used to look at the biochemical recurrence-free survival (bRFS) between the group’s differences. The sensitivity and specificity of survival prediction were examined by subject operating characteristic (ROC) analysis. The area under the ROC curve (AUC) was calculated as an indicator of predictive accuracy and principal component analysis (PCA) was performed to explore the distribution between groups.

### The construction of the nomogram

Univariate and multivariate cox regression analyses were performed on the entire cohort of TCGA patients to determine whether the predictive effect of lipid metabolism-related prognostic models was independent of clinical variables. A biochemical recurrence model, Nomogram, was developed using R package rms to predict biochemical recurrence-free survival in prostate cancer patients at 1, 3, and 5 years after radical surgery based on differential lipid metabolism genes and clinical data independent of prognostic correlations. Calibration plots were drawn for further evaluation of the discriminatory power of the Nomogram. 45° lines represent the best prediction.

### Gene pool enrichment analysis

Download Genomic Enrichment Analysis (GSEA) is used to identify pathways that are primarily enriched between high and low-risk groups.

### Immune cell infiltration

The CIBERSORT algorithm was used to analyze the immunological characteristics of the high-risk and low-risk populations in the TCGA cohort.

## Results

### Preliminary screening and identification of differential genes related to lipid metabolism

**Figure 1:**
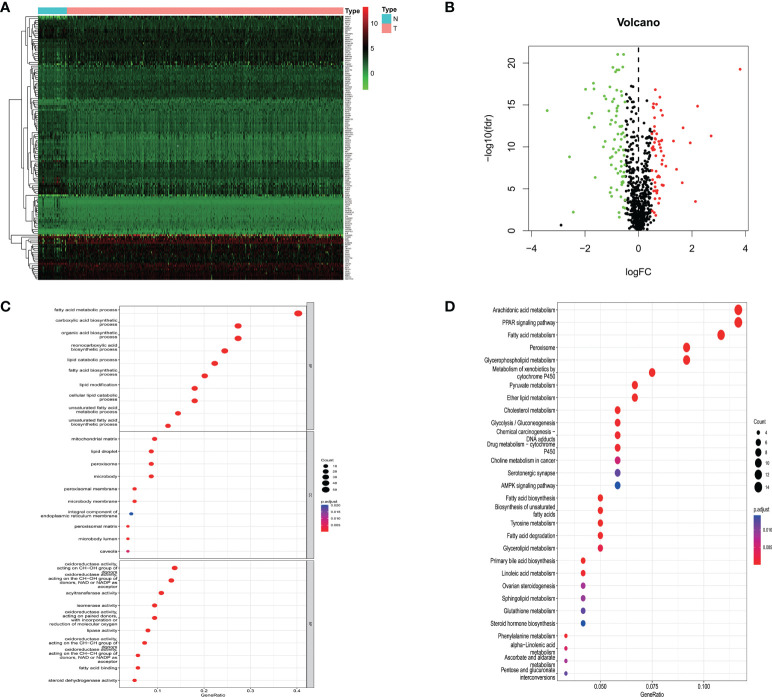
**(A)** Heatmap showing lipid metabolism-related genes differentially expressed in prostate cancer tissue and normal prostate tissue; **(B)** Volcano map of lipid metabolism-related differential genes; **(C)** GO analysis of related differential genes including biological processes, cellular components and molecular functions; **(D)** KEGG analysis of differential genes.

540 mRNA samples (489 prostate cancer tissues and 51 non-tumor tissues) were analyzed in the TCGA database. By Wilcoxon signed-rank test, 139 differentially expressed lipid metabolism-related genes were obtained, of which 62 genes were up-regulated and 77 genes were down-regulated in prostate cancer tissues (**Figures 1A, B**).

### Functional enrichment of differential genes related to lipid metabolism

To further elucidate the potential mechanisms of lipid metabolism-related differential genes, a functional enrichment analysis of 139 differential genes was performed. Among the biological processes, lipid metabolism-related differential genes were mainly enriched in fatty acid metabolism, carboxylic acid biosynthesis, organic acid biosynthesis, monocarboxylic acid biosynthesis, and lipid catabolism. Among the cellular components, they are mainly found in the mitochondrial matrix, lipid droplets, peroxisomes, microsomes, and peroxisomal membranes. Among the molecular functions, the main enrichment is in oxidoreductase activity, acyltransferase activity, isomerase activity, lipase activity, and fatty acid-binding. In the KEGG pathway, the results showed that the differential genes were mainly enriched in arachidonic acid metabolism, PPAR transduction signaling pathway, fatty acid metabolism, peroxisomal, and glycerophospholipid metabolism. These results above suggest that lipid metabolism may be involved in the development of prostate cancer (**Figures 1C, D**).

### PPI network

**Figure 2:**
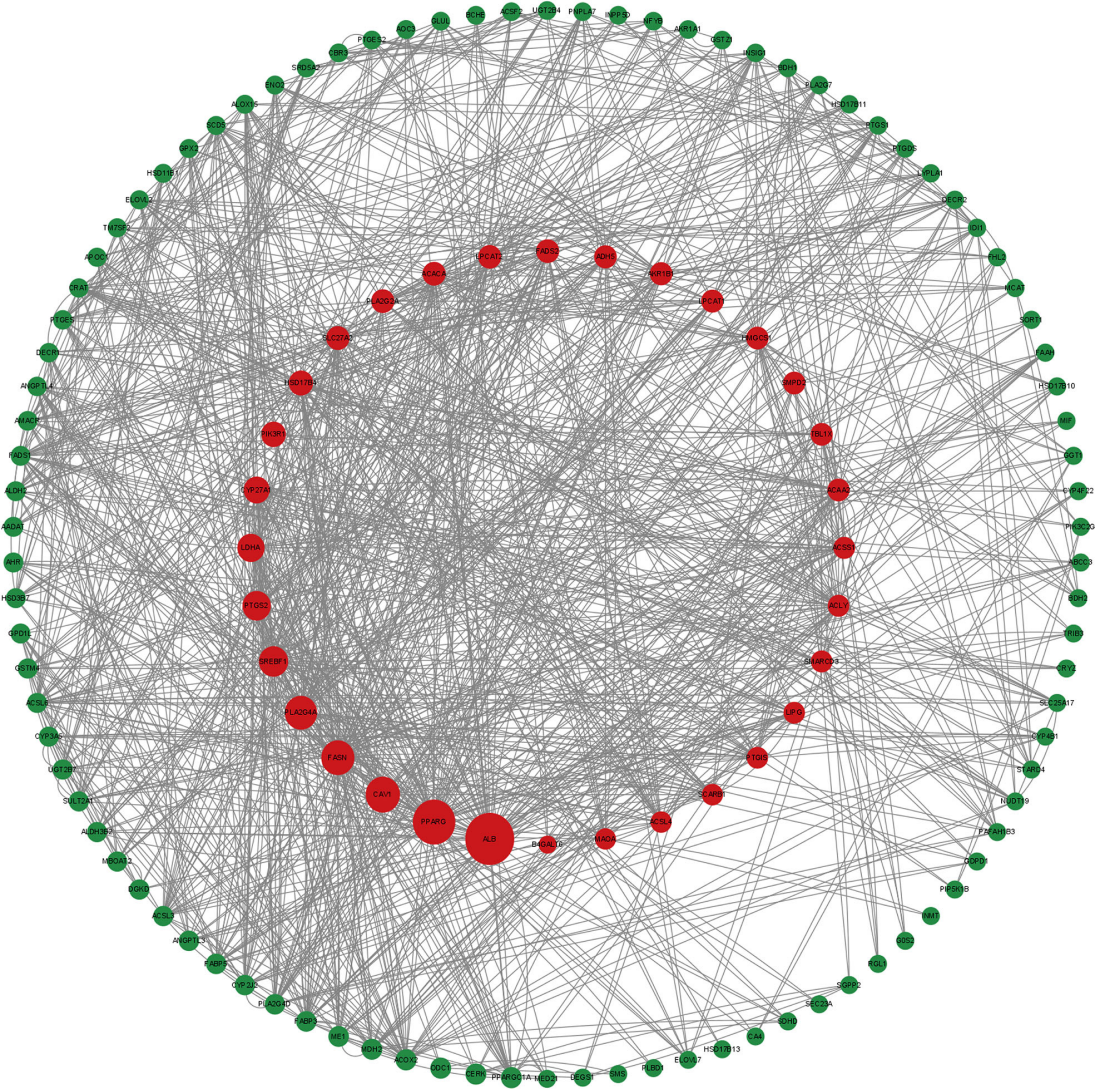
Differential gene Protein-protein interaction (PPI) network.

To study the association of differentially expressed lipid metabolism-related genes, a PPI network containing 126 nodes and 574 edges was built and visualized by Cytoscape (**Figure 2**).

### Construction and validation of a genetic model of lipid metabolism associated with biochemical relapse

**Figure 3:**
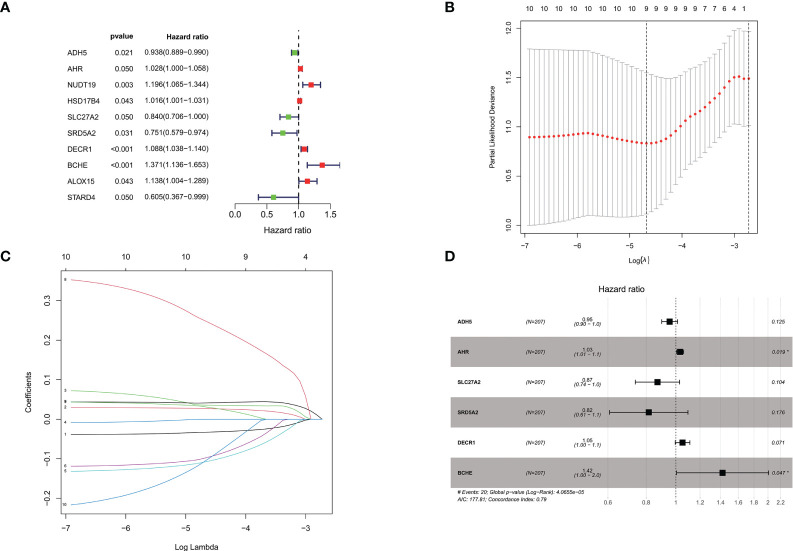
**(A)**. Univariate cox analysis of the TCGA cohort training group screened the forest plot of differential genes associated with BCR; **(B, C)** 9 genes were further screened by LASSO regression analysis; **(D)**. Multivariate cox regression analysis was performed to finally screen the forest plot of 6 genes associated with BCR. *P < 0.05.

In the training group of the TCGA prostate cancer patient cohort, a one-way cox regression analysis was performed on the 139 differential genes listed above to identify differential genes associated with biochemical recurrence-free survival in prostate cancer. A total of 10 differential genes associated with biochemical recurrence were identified by univariate cox analysis. Subsequently, to ensure stability and feasibility, we further screened 9 genes associated with biochemical recurrence in prostate cancer patients by LASSO cox regression analysis. Finally, six genes, including ADH5A, AHR, SLC27A2, SRD5A2, DECR1, and BCHE, were selected by multivariate cox regression analysis. We then developed a genetic signature-based risk score based on the cox coefficients of these six genes, as follows. (**Figure 3**).


Risk score = ADH5*−0.04662+AHR*0.03228+SLC27A2*−0.13818+SRD5A2*−0.20324+DECR1*0.05072+BCHE*0.35147

Based on the risk score, patients in the training group were divided into a high-risk group and a low-risk group. Kaplan-Meier survival curves showed that in the training group, prostate cancer patients in the high-risk group had a lower model of recurrence-free survival than those in the low-risk group. Applying the same regression coefficients and algorithms to the TCGA internal validation set and MSKCC validation set respectively, the results were as expected, with higher recurrence-free survival rates in the low-risk group.

**Figure 4:**
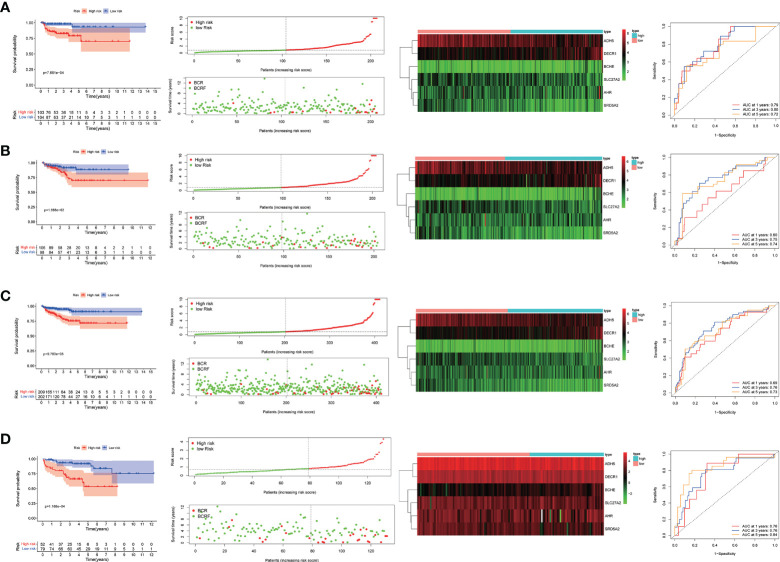
**(A–D)**. Predictive value of genetic characteristics for biochemical relapse (BCR) in each cohort. risk scores were significantly higher in patients with BCR disease than in patients without BCR disease. Kaplan-Meier analysis showed that relapse-free survival (RFS) was significantly longer in patients with low risk scores than in patients with low risk scores. Time-related subject operating curve (ROC) analysis showed that each group predicted 1-, 3-, and 5-year bRFS.

In addition, as shown by the time-dependent ROC analysis, the 1-, 3- and 5-year AUC values for the training set were 79%, 80%, and 72%, respectively; the 1-, 3- and 5-year AUC values for the internal validation set were 60%, 75%, and 74%; the 1-, 3- and 5-year AUC values for the entire TCGA cohort were 69%, 76%, and 73%; and in the MSKCC external validation set, the 1-, 3- and 5-year AUC values were 76%, 76%, and 84%. This indicates that the risk assessment model we developed has the good predictive capability (**Figure 4**).

**Figure 5:**
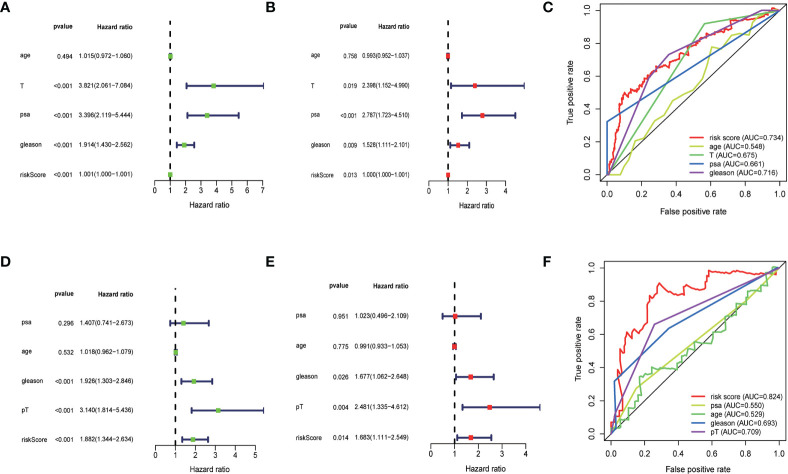
**(A, B)** Univariate and multivariate Cox regression analysis of the TCGA cohort. **(D, E)** Univariate and multivariate cox regression analyses for the MSKCC cohort. **(C, F)** time-dependent ROC analyses.The results showed that the risk score had significant prognostic value.

To determine whether the risk scores generated by the prognostic model were independent factor compared to other clinicopathological factors, we performed univariate and multivariate cox regression analyses on the TCGA cohort and the MSKCC cohort. The results showed that this lipid metabolism-related predictive model was an independent predictor of the biochemical recurrence of prostate cancer (TCGA: unicox: P<0.001, multicox:P=0.013;MSKCC: unicox: P<0.001, multicox:P=0.014).To estimate the prognostic power of the lipids signature, the time-dependent ROC analyses were used, and the area under curve (AUC) values were calculated. We found that the average AUC value of the risk score for RFS was 0.778 at five years follow-up in the TCGA set, which was significantly higher than those of GS (AUC = 0.716), PSA (AUC = 0.661) and pT (AUC = 0.675). Similarly, in the MSKCC set, average AUC value of the risk score (AUC = 0.824) was also considerably higher than those associated with GS (AUC = 0.693) and pT (AUC = 0.709). The outcomes mentioned above suggested that the lipid-metabolism signature served as a more powerful predictor for BCR than other clinical features in localized PCa patients (**Figure 5**).

### Construction and validation of nomograms

**Figure 6:**
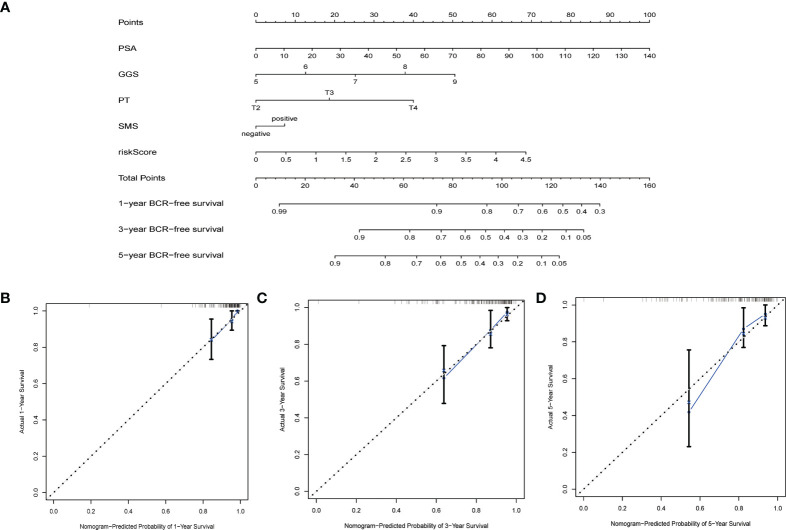
Nomograms and calibration plots based on the characteristics and clinical variables we established. **(A)** Probabilistic nomogram for predicting 1-, 3-, and 5-year biochemical recurrence-free survival in patients with localized prostate cancer; **(B–D)** Columnar line plot calibration plots for consistency tests between 1-, 3-, and 5-year survival predictions and actual outcomes.

By integrating genetic features with clinical parameters (PSA, GS, pT, SMS) in the MSKCC cohort, we constructed a nomogram to visualize the predicted risk of biochemical recurrence. The results showed that the line plot yielded AUCs of 76.08%, 76.18%, and 83.66% at the 1-year, 3-year, and 5-year biochemical recurrence-free survival time points, respectively. The calibration curves showed that actual survival rates were similar to their survival rates (**Figure 6**).

### Genome enrichment analysis

**Figure 7:**
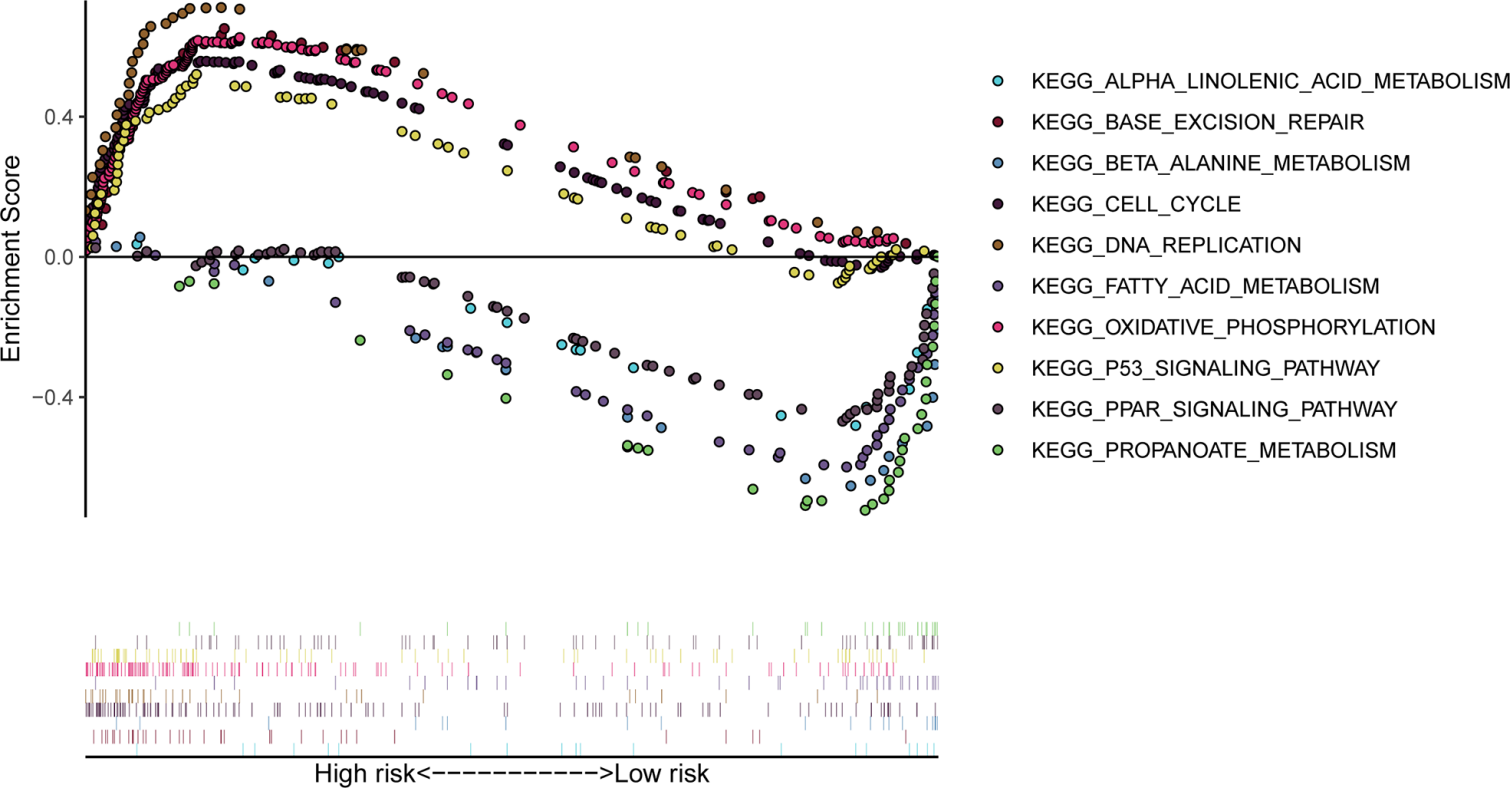
GSEA significantly enriched the KEGG pathway in the TCGA cohort. Above the horizontal axis indicates pathways in the high-risk group and below the horizontal axis indicates pathways in the low-risk group.

The genomic enrichment analysis software (GSEA) was used to identify significantly enriched KEGG pathways in the TCGA whole-group cohort. Results showed that basal excision repair, DNA replication, cell cycle, p53 signaling pathway, and oxidative phosphorylation were significantly enriched in the high-risk group; propionate metabolism, β_alanine_metabolism, fatty acid metabolism, PPAR signaling pathway, and α_linolenic_acid_metabolism were significantly enriched in the low-risk group (**Figure 7**).

### Immunological infiltration analysis

**Figure 8:**
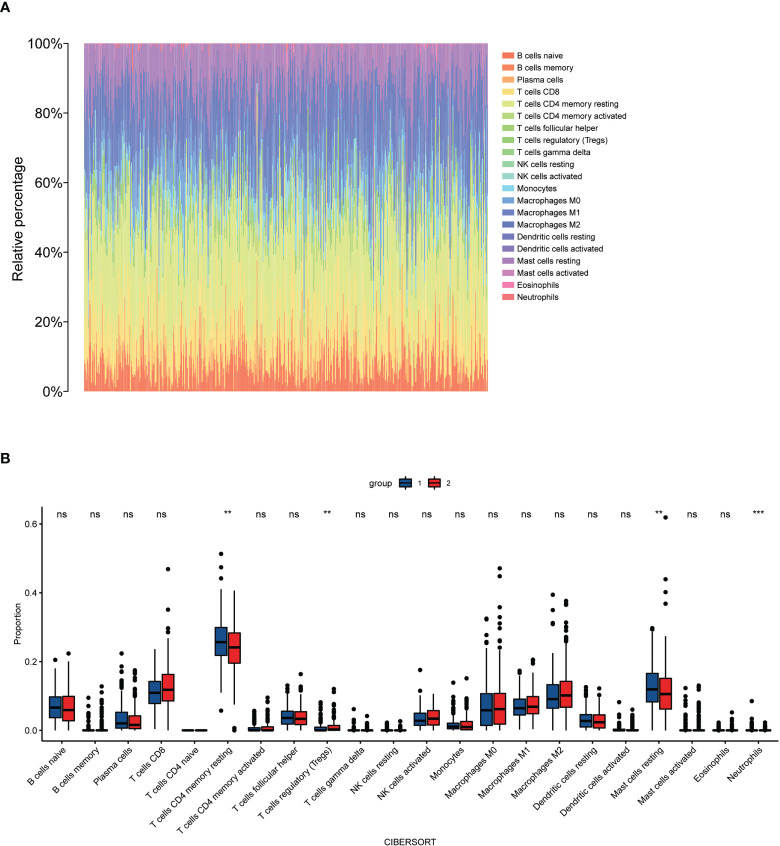
Immune cells infiltrate between high-risk and low-risk populations. **(A)** CIBERSORT immune cell ratio map; **(B)** Differential analysis of immune cells in different risk populations. **P < 0.01, ***P < 0.001, ns, P>0.05.

To investigate the relationship between high and low-risk groups and immune infiltration in the TCGA cohort, we analyzed a pooled set of 22 immune cell phenotypes using CIBERSORT. According to the results, Tregs infiltrated more in the high-risk group than in the low-risk patients, while resting CD4 cells, resting mast cells, and neutrophils were more activated in the low-risk group than in the high-risk group. This suggests that Tregs are an important predictor of poor prognosis for biochemical recurrence of prostate cancer (**Figure 8**).

## Discussion

Prostate cancer is the most common malignancy in men and is the fifth leading cause of cancer death (1). Although early-stage limited prostate cancer can be effectively treated with radical surgery, approximately 27% to 53% of prostate cancer patients still develop local recurrence or distant metastases within 10 years of surgery (15). Biochemical recurrence is a determinant risk factor for distant metastases and overall mortality in prostate cancer, with approximately 30% of patients experiencing clinically manifest distant metastases in the absence of secondary treatment following biochemical recurrence and 19% to 27% likely to suffer prostate cancer-specific death at 10 years (16). There is therefore a great need to effectively evaluate patients with prostate cancer who are at increased risk of biochemical recurrence. There has been a growing body of research to identify biomarkers to improve the prediction of biochemical recurrence of prostate cancer. Although many genetic signatures have been established to predict prostate cancer biochemical recurrence after RP, prognostic genetic markers based on lipid metabolism have not been reported in prostate cancer biochemical recurrence. Furthermore, given the important role of lipid metabolism in cancer progression and development, we wanted to explore the possibility of establishing a robust lipid metabolism-based genetic marker to predict the biochemical recurrence of prostate cancer.

In this study, multi-step bioinformatics analysis was performed to identify a gene signature based on lipid metabolism that predicts biochemical recurrence-free survival in prostate cancer patients. First, 139 differential genes were obtained by analyzing the expression of lipid metabolism in prostate cancer tissue and comparing it to normal prostate tissue in the TCGA dataset. The biological functions of differential genes associated with lipid metabolism in prostate cancer were then investigated by PPI network and functional enrichment analysis. GO and KEGG analyses indicated that differential genes were involved in arachidonic acid metabolism, PPAR transduction signaling pathway, fatty acid metabolism, peroxisome, and glycerophospholipid metabolism. Subsequently, we randomly divided the TCGA cohort into training and internal validation groups and used the MSKCC cohort as the external validation set. Based on the training group, we performed initial screening by univariate cox analysis, followed by lasso regression analysis and multivariate cox analysis, and finally constructed six gene prognostic signatures associated with lipid metabolism. bRFS analysis showed that the signature was a good predictor of biochemical recurrence of prostate cancer. Univariate and multivariate cox regression analyses revealed that the calculated risk score was an independent risk factor for the biochemical recurrence of prostate cancer. In addition, the signature was strongly associated with poorer clinical features, including pathological T-stage, PSA, and Gleason score. We used time-correlated ROC analysis and the results showed that our model exhibited good predictive performance on both the training and validation datasets. Including Nomogram analysis shows that the model combined with clinical indicators can be used to accurately predict biochemical recurrence of prostate cancer and can provide clinicians with a quantitative tool to predict biochemical recurrence and improve risk stratification, facilitating better-individualized treatment for patients. With the help of nomogram and clinical data of patients, patients with high risk of biochemical recurrence can be further risk stratification, so that patients with higher total scores can be more closely monitored actively. At the same time, patients with Gleason score>8 or pathological stage>T3 were treated with salvage radiotherapy as early as possible before serum PSA>0.2ng/ml, or even combined with ADT, so as to avoid further recurrence and metastasis of the tumor and to achieve a longer relapse free survival (17, 18).

GSEA analysis showed that patients in the high-risk group were associated with oxidative phosphorylation and the p53 signaling pathway, while patients in the low-risk group were associated with propionate metabolism, beta_alanine_metabolism, fatty acid metabolism, the PPAR signaling pathway, and the alpha_linolenic_acid_metabolism pathway.

In addition, through the CIBERSORT algorithm, our signature identified an association with increased numbers of Treg cells in patients with high-risk scores. Tregs can promote tumor progression by suppressing effective anti-tumor immunity and reducing the benefits of immunotherapy (19). Flammiger, A found Tregs to be associated with patients with advanced PCa, suggesting that these cells contribute to a poor clinical disease course (20). In conclusion, our findings suggest that Treg cells may represent a new target for PCa therapy.

Some of these six genes that make up our signature are associated with cancer, including prostate cancer. adh5 is the gene encoding GSNOR and is located on the reverse strand of chromosome 4 (4q23 - chr4: 999993567-10000985), and high levels of human epidermal growth factor receptor 2 (HER2) expression in breast tumors are associated with low GSNOR expression and apoptosis protein S-nitrosylation was associated with an increase in (21). This study also determined that increased GSNOR expression in HER2 tumors was associated with higher patient survival. The aryl hydrocarbon receptor (AHR) is the only member of the basic helix-loop-helix-PER-ARNT-SIM (bHLH-PAS) subgroup of the bHLH transcription factor superfamily known to be ligand-activated to mediate the action of the carcinogenic 2,3,7,8-tetrachlorodibenzo-p-dioxin (2,3,7,8-tetrachlorodibenzo-p-dioxin). Commonly referred to as “dioxin”), is known for its toxicity and tumor-promoting properties (22). AhR was found to be constitutively active in advanced prostate cancer cell lines that mimic hormone-refractory prostate cancer, and increased nuclear AHR localization was positively associated with higher tumor grade, poor differentiation, and/or poor prognosis (23). TCDD also induced Tregs cell production, suggesting that the presence of endogenous AHR ligands enhances cell production in the T vehicle registry tumor microenvironment, which is consistent with an increase in Tregs cells in the high-risk group for biochemical recurrence compared to the low-risk group in our study (24). SLC27A2 has been reported to regulate the tumor suppressor gene PARP, with reduced levels of SLC27A2 expression in metastatic tumors compared to non-metastatic neuroendocrine tumors (25). In addition, upregulation of SLC27A2 promotes proliferation and invasion of differentiated thyroid cancer cells (26). 5-alpha reductase type 2 (i.e. SRD5A2) allelic variants occur with the highest frequency in African American men and are associated with increased risk of PC and are the only individual biomarker significantly associated with 5-year risk of metastatic disease (27, 28). 2,4-dienoyl coenzyme A reductase (DECR1), a beta-oxidation coenzyme, is a clinically relevant biomarker for CRPC, and DECR1 deficiency impairs lipid metabolism and reduces CRPC tumor growth (29). What’s more, DECR1, encoding the rate-limiting enzyme for oxidation of polyunsaturated fatty acids (PUFAs), caused cellular accumulation of PUFAs, enhanced mitochondrial oxidative stress and lipid peroxidation, and induced ferroptosis. DECR1 knockdown selectively inhibited β-oxidation of PUFAs, inhibited proliferation and migration of PCa cells and the overexpression of DECR1 is associated with shorter relapse-free survival (30). Butyrylcholinesterase (BChE) is an alpha-glycoprotein found in the nervous system and liver. Studies have shown that a decrease in cholinesterase activity and the consequent increase in acetylcholine may lead to cholinergic hyperstimulation and increase cell proliferation in PCa, and moreover it has been shown that patients with increased preoperative BCHE levels have a higher 5-year BRRFS rate and are an independent prognostic factor for PCa after RP (31, 32).

### Study strengths and limitations

The main strength of this study is the construction and validation of a prognostic signal based on genes related to lipid metabolism that is strongly associated with biochemical recurrence in prostate cancer patients. The main limitation of this study is the lack of *in vivo* and *in vitro* experimental validation. Therefore, further experiments are needed to validate the function of lipid metabolism-related genes in prostate cancer.

## Conclusion

In conclusion, we performed a comprehensive study by bioinformatics analysis to develop a six-gene signature based on lipid metabolism to predict BCR-free survival in prostate cancer patients after RP. The higher the risk score, the higher the probability of BCR. And, the signal also suggests that Tregs are associated with the risk of biochemical recurrence progression of prostate cancer and thus can be used as new potential therapeutic targets. Furthermore, the genes contained in our signal should be investigated to better understand the molecular mechanisms of prostate cancer development and progression.

## Data availability statement

Publicly available datasets were analyzed in this study. This data can be found here: The original data of this study came from TCGA research network (https://www.cancer.gov/tcga) Cbioportal database (https://www.cbioportal.org/), KEGG (https://www.kegg.jp/kegg/pathway.html), and msigdb (https://www.gsea-msigdb.org/gsea/msigdb/), they are publicly available databases.

## Author contributions

YC, project development, data analysis, and manuscript writing. JL, data collection and analysis. All authors contributed to the article and approved the submitted version.

## Conflict of interest

The authors declare that the research was conducted in the absence of any commercial or financial relationships that could be construed as a potential conflict of interest.

## Publisher’s note

All claims expressed in this article are solely those of the authors and do not necessarily represent those of their affiliated organizations, or those of the publisher, the editors and the reviewers. Any product that may be evaluated in this article, or claim that may be made by its manufacturer, is not guaranteed or endorsed by the publisher.
